# Supplementary material for: Poor glycemic control, cardiovascular disease risk factors and their clustering among patients with type 2 diabetes mellitus: A cross-sectional study from Nepal
Source: PLoS One. 2022 Jul 25;17(7):e0271888. doi: 10.1371/journal.pone.0271888 (PMC9312399; doi:10.1371/journal.pone.0271888)
Supplement: Attachment — Submitted filename: Peer-review to PLoS One.docx [file pone.0271888.s004.docx]

**Title: Poor glycemic control, cardiovascular disease risk factors and their clustering among patients with type 2 diabetes mellitus: A cross-sectional study from Nepal**

**Dear Editor,**

I have gone through the manuscript and found it well drafted. The scientific rigor and other technical issues are generally acceptable based on my experience in the field.

However, I need some clarifications or rebuttal for the following points:

**Abstract: Method**

- Poisson logistic regression vs Poisson regression: Which one is generally acceptable to use?

**Introduction,**

- Instead of using the term ‘recent years’, ‘latest’ etc., I recommend the researchers to provide the exact dates/years for readers several years later. Update this section with latest citations as well

**Methods**

- Include study period in the subtitle (rename as ‘study design, settings and period’)
- …….confirmed diagnosis of T2DM, who were diagnosed at least one month before data collection……Why did you use one month as time limit?
- “……T2DM was confirmed from the prescription card of the patient. ………..We selected patients with T2DM from randomly selected centers and then they were invited to visit the Provincial Ayurveda Hospital….”. Which one was applied initially? You contacted the patient based on the card as tracer or contacted patient then back to the card for confirmation. Clarify
- Sample size and sampling 🡪 sample size determination and sampling procedure
- It that a simple or multistage cluster sampling?
- In simple cluster sampling, all units in the selected cluster should be studied, whereas in multistage, double sampling procedures for settings and individuals should be done.
- Page 7: line 124 ……to select representative samples from all kinds of health centers 🡪 rewrite as health facilities
- After collecting socio-demographic, anthropometric and, related variables, you invited the patient to visit the hospital with eight hours of fasting. What did you do when the patient missed/overlooked your invitation?
- …….We asked patients to provide blood for postprandial blood sugar testing after two hours of their usual meal…….This might be tiresome for the patients as this was the third time to contact investigators. This part would have been collected when you collected the sociodemo….related variables just to measure Random blood sugar. If the intention was to measure oral glucose tolerance test (OGTT), the investigator him/herself should provide oral glucose (fixed dose) two hours earlier.
- Mixed up of definition of terms with description of variables
- “We defined poor glycemic control as fasting blood sugar >130 milligrams per deciliter (mg/dl) and/or postprandial blood sugar ≥180 mg/dl [8]”. Many studies available online,however, used a cut-off point 126 mg/dl for FBS and 200 mg/dl for RBS. Provide a justification for it.
- The biochemical variables should be clearly defined again with clear cut-off points and in line with the universal standard (needs separate operational definitions)
- **Page 10: Line 211-212: ….yes (1)** if clustering of four or more risk factors and/or poor glycemic control were present, and no (0): if fewer than three risk factors were present….. What about three?
- **Page 10: Line 214-215**: Socio-demographic and clinical variables were entered into simple and multiple logistic regression models to determine crude and adjusted odds ratios of clustering of CVD risk factors, respectively. Simple and multiple logistic regression 🡪 Bivariable and multivariable BINARY logistic regression? Clarify if it is to mean different from this.

**Generally,**

- Scientific procedures in method section shall be preferentially written in passive voice. Almost all the entire manuscript was written in active voice.
- The active voice is direct, clear and concise and the passive voice is indirect, but sometimes it is useful to emphasize the research, instead of the researcher in scientific world.
- I left this part to the discretion of the research to provide justification.
- I recommend the researchers to use the balance between the two forms.
