# Supplementary material for: Poor glycemic control, cardiovascular disease risk factors and their clustering among patients with type 2 diabetes mellitus: A cross-sectional study from Nepal
Source: PLoS One. 2022 Jul 25;17(7):e0271888. doi: 10.1371/journal.pone.0271888 (PMC9312399; doi:10.1371/journal.pone.0271888)
Supplement: Attachment — Submitted filename: Response to reviewer 1.docx [file pone.0271888.s005.docx]

Review by Reviewer 1:

The authors examined the prevalence of poor glycemic control, CVD risk factors, and their clustering among patients with T2DM and concluded that the majority of the patients with T2DM had poor glycemic control and CVD
risk factors in the city Tulsipur in Nepal. Needs for the policies and programs focused on the prevention and better management of T2DM and CVD risk factors were rised.

This is a very well conducted study and written paper on a very important topic.
I do not see any major flaw.

Dear Reviewer,

Thank you for providing valuable insight about our manuscript.
